# Supplementary material for: Thermal adaptation of soil microbial growth traits in response to chronic warming
Source: Appl Environ Microbiol. 2023 Oct 25;89(11):e00825-23. doi: 10.1128/aem.00825-23 (PMC10686086; doi:10.1128/aem.00825-23)
Supplement: Fig. S1 — Ratkowsky 1983 fits for all isolates. [file aem.00825-23-s0001.pdf]

Fig S1. Ratkowsky 1983 model was fitted on data of growth rate over temperature for each isolate. Temperature sensitivity of growth, optimum growth temperature, and maximum growth temperature were estimated from model fits and used for phylogenetic group comparisons.

19YEA23

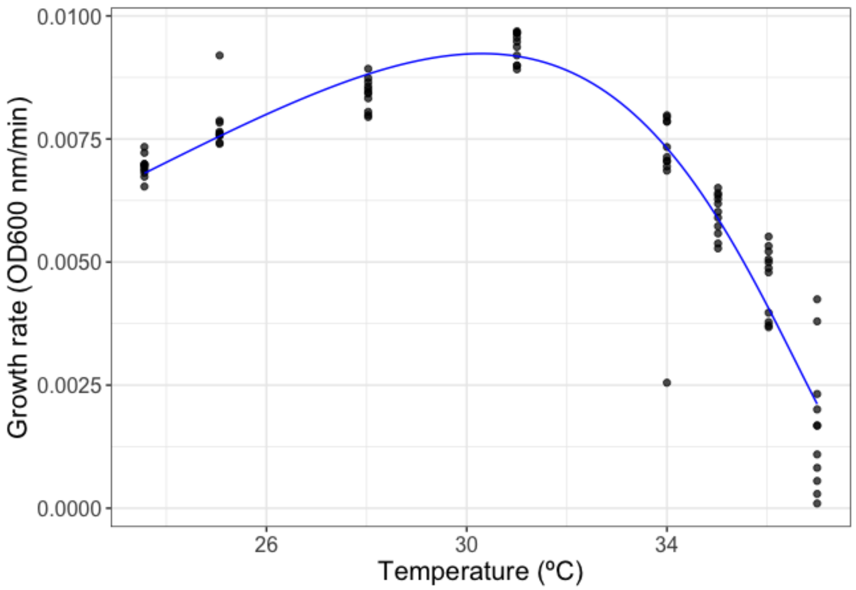

24YEA27

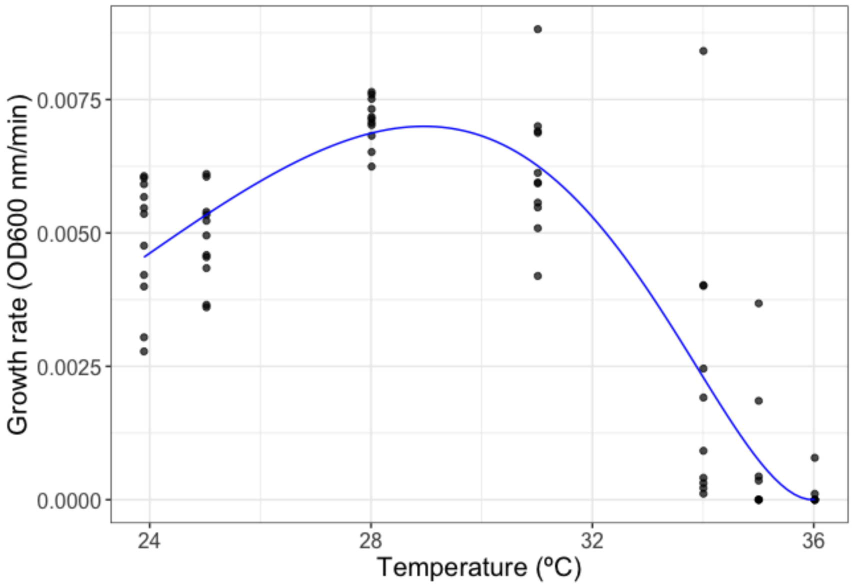

28DA2

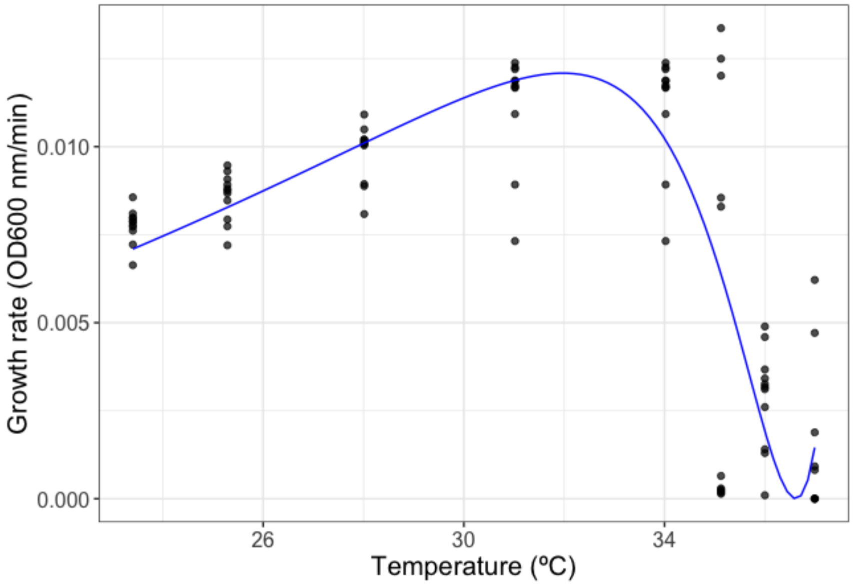

28YEA48

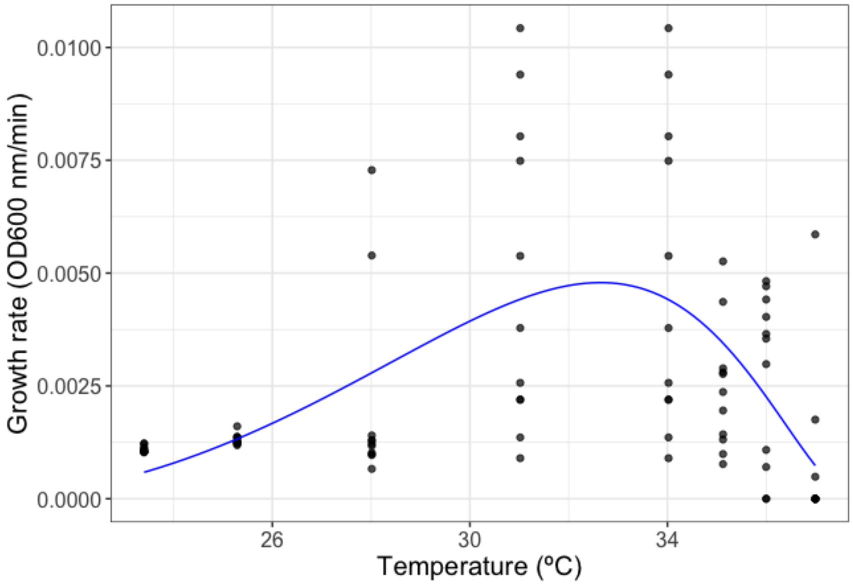

AN5

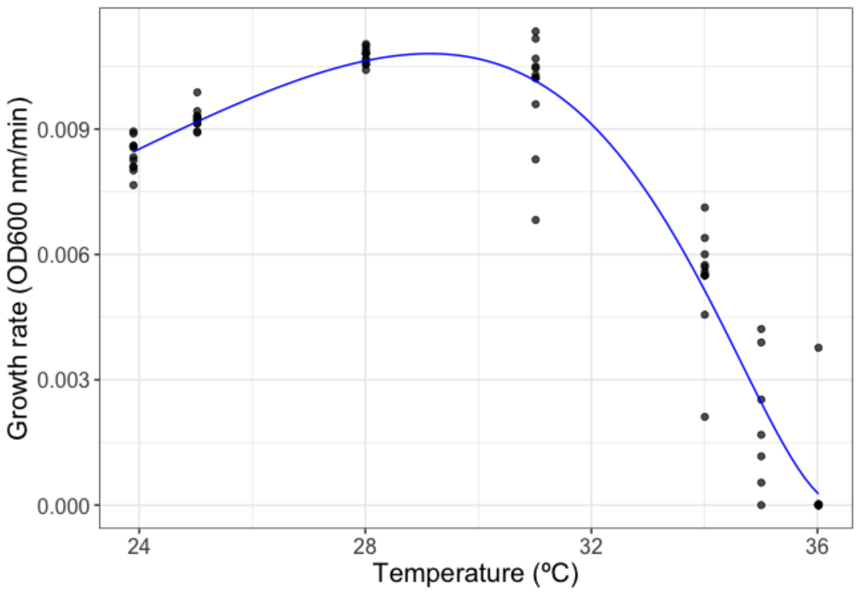

Growth rate (OD600 nm/min)
0.000
0.003
0.006
0.009
24
28
32
36
Temperature (ºC)

AN6A

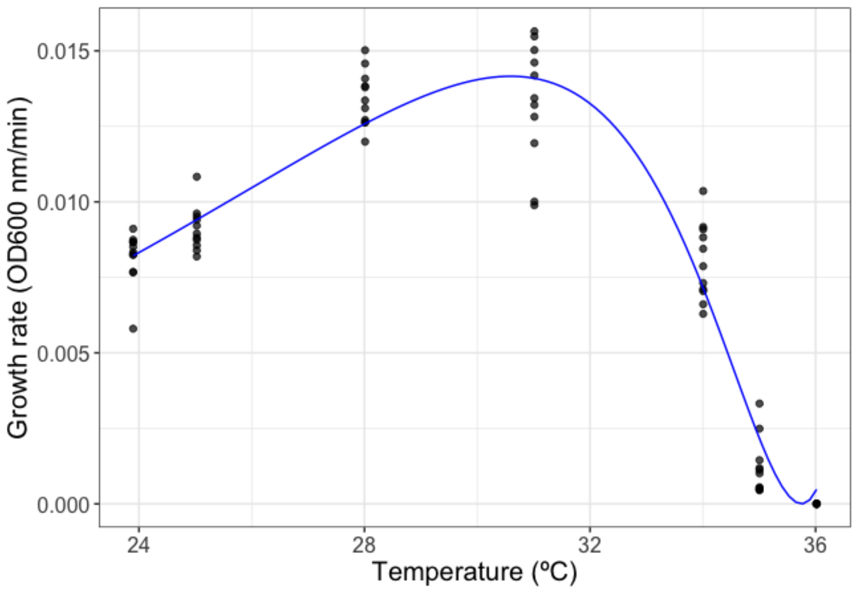

Growth rate (OD600 nm/min)
0.000
0.005
0.010
0.015
24
28
32
36
Temperature (ºC)

AN63

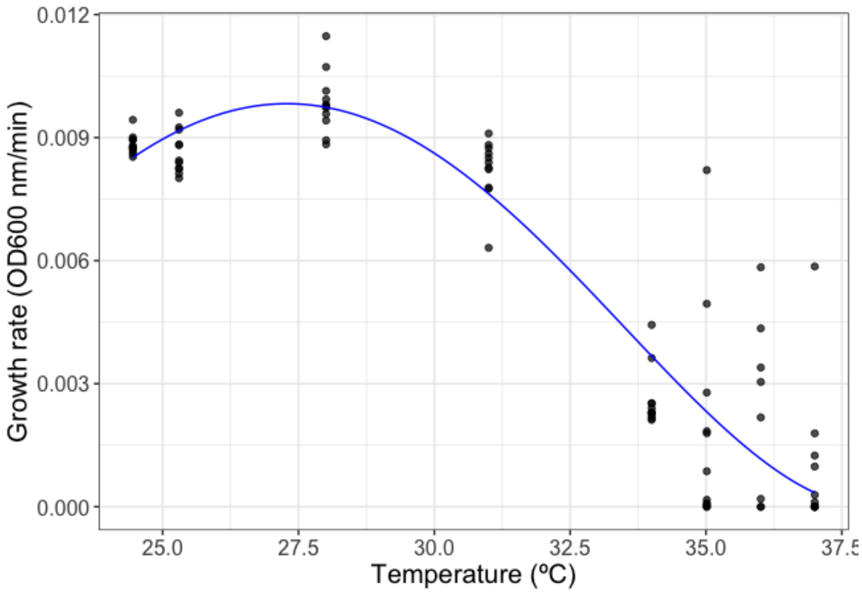

0.012
0.009
0.006
0.003
0.000
Growth rate (OD600 nm/min)
25.0
27.5
30.0
32.5
35.0
37.5
Temperature (ºC)

AN64

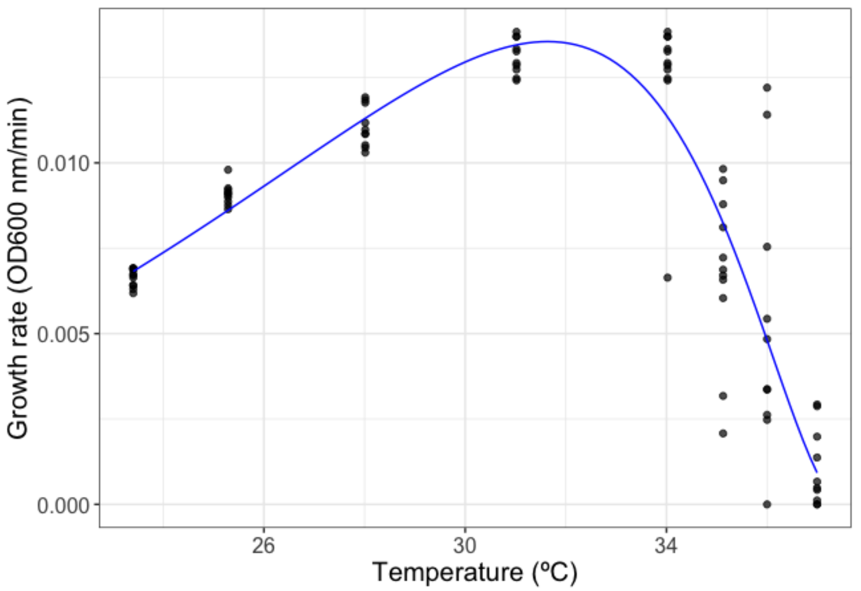

0.010
0.005
0.000
Growth rate (OD600 nm/min)
26
30
34
Temperature (ºC)

AN67

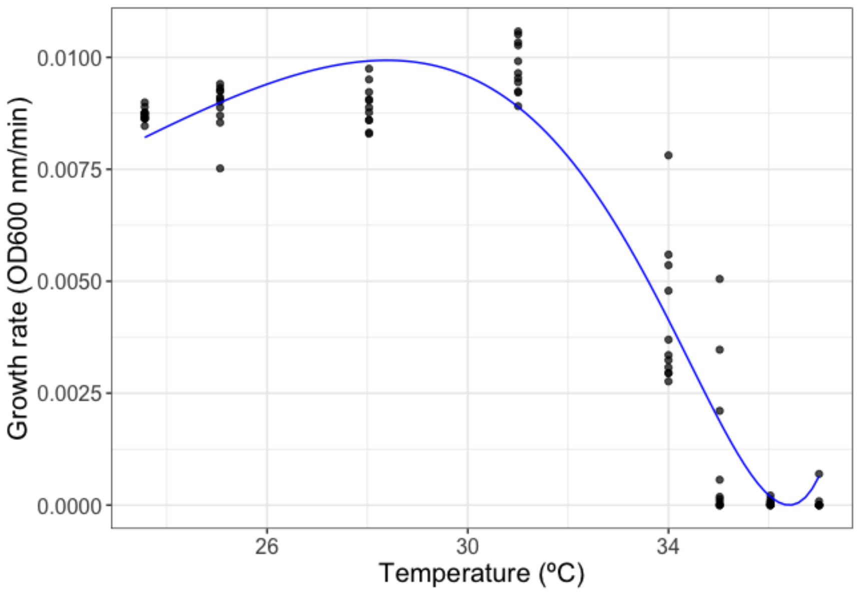
0.0100
0.0075
0.0050
0.0025
0.0000
Growth rate (OD600 nm/min)
26
30
34
Temperature (ºC)

AN68

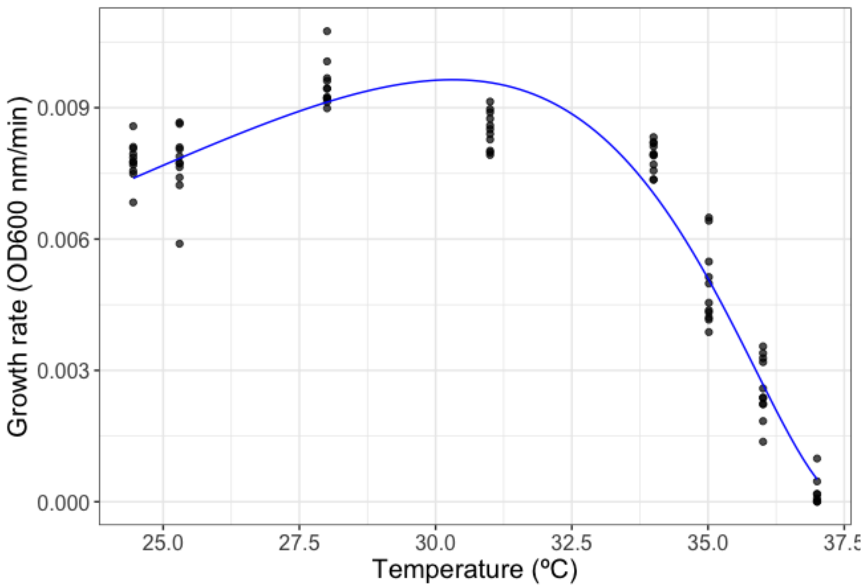
0.009
0.006
0.003
0.000
Growth rate (OD600 nm/min)
25.0
27.5
30.0
32.5
35.0
Temperature (ºC)

AN69

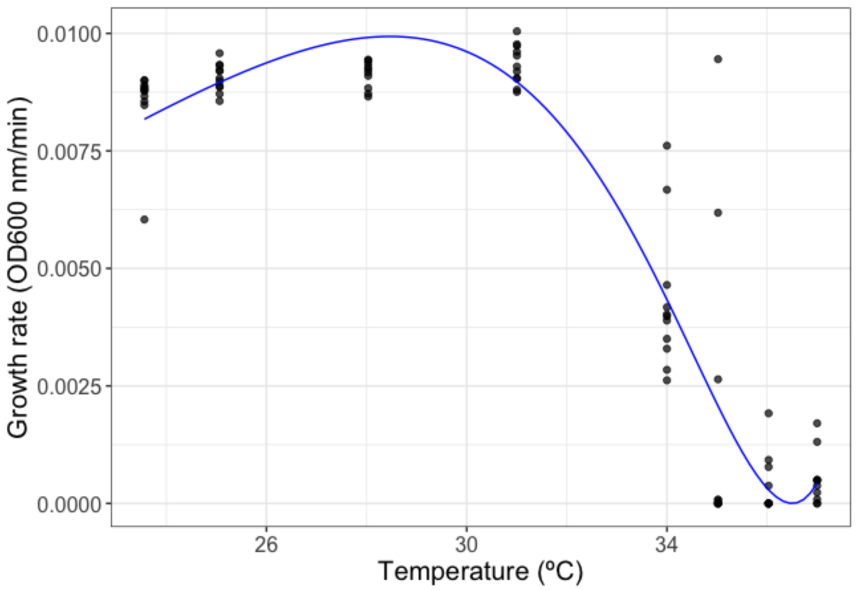

AN70

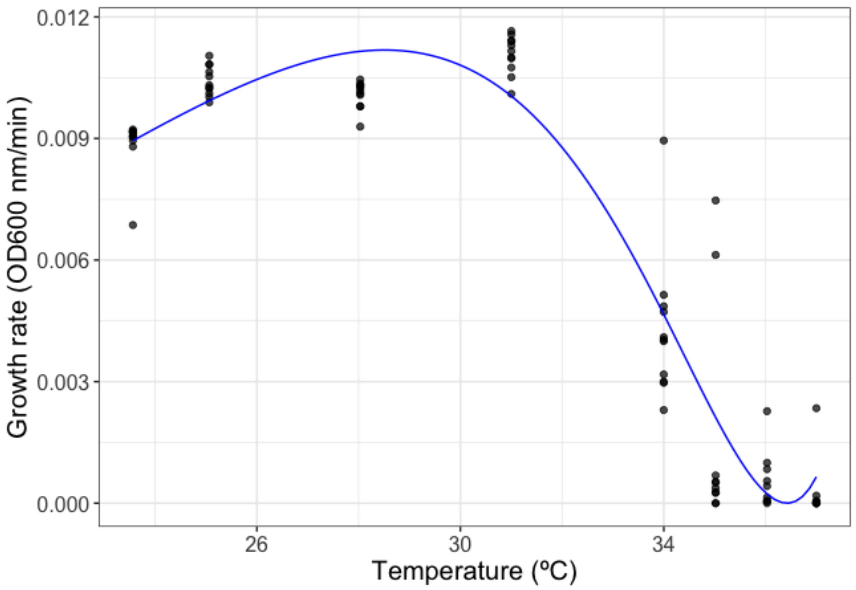

AN73

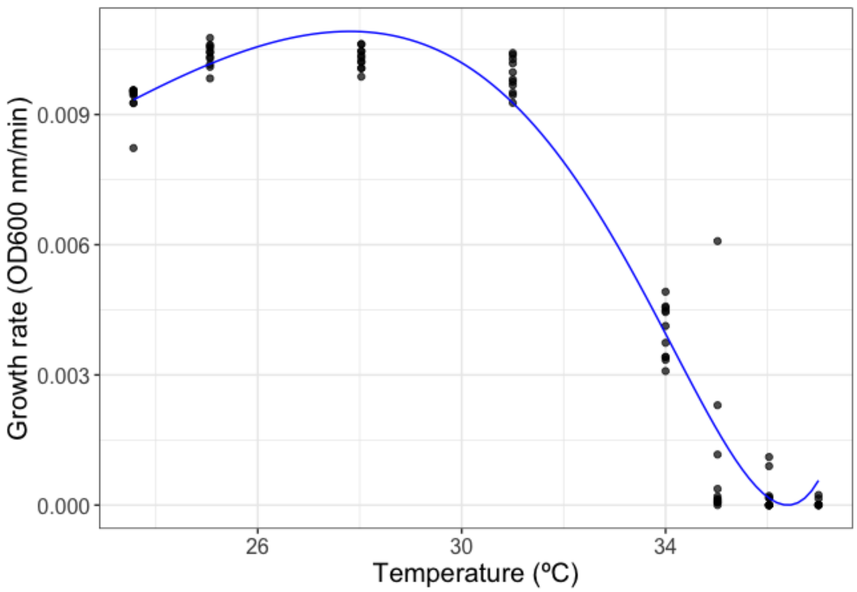

Growth rate (OD600 nm/min)
0.009
0.006
0.003
0.000
26
30
34
Temperature (ºC)

AN78

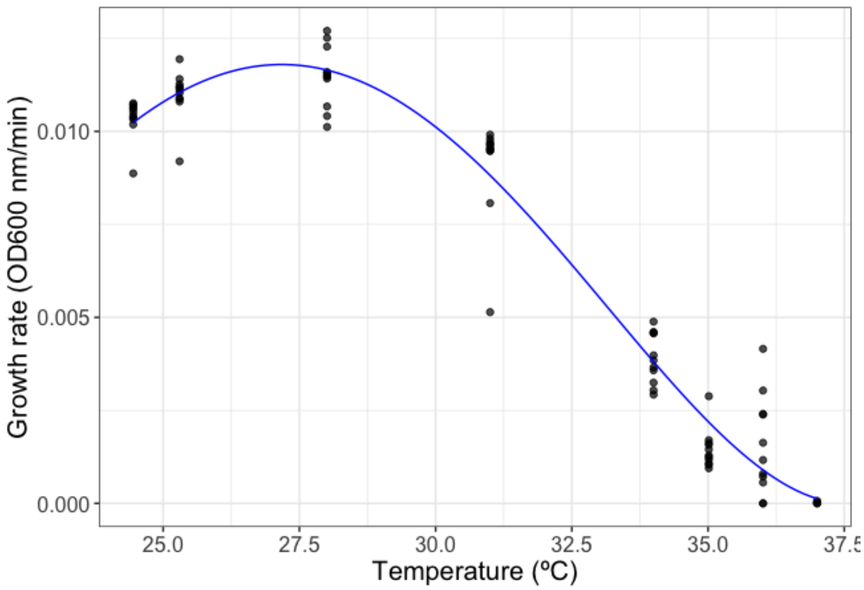

Growth rate (OD600 nm/min)
0.010
0.005
0.000
25.0
27.5
30.0
32.5
35.0
Temperature (ºC)

AN80A

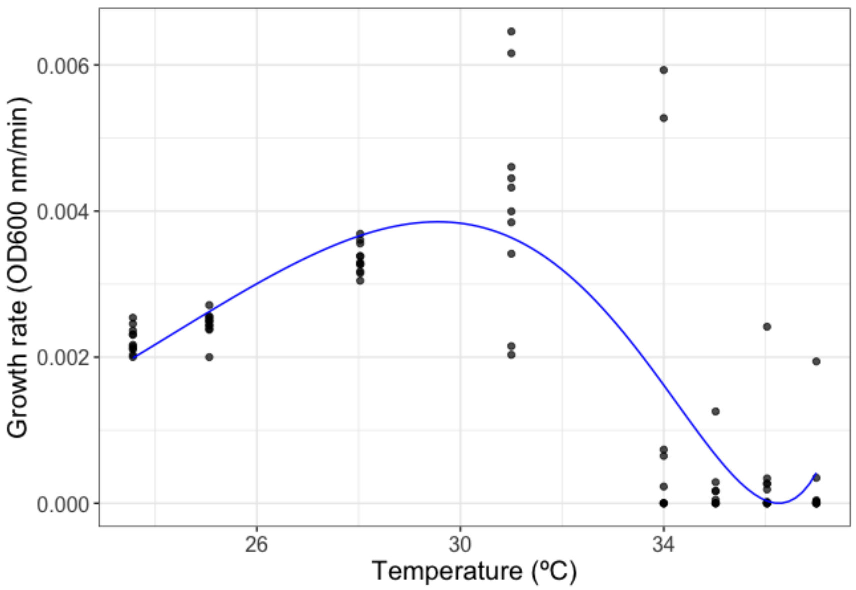
Growth rate (OD600 nm/min)
0.006
0.004
0.002
0.000
26
30
34
Temperature (ºC)

AN83

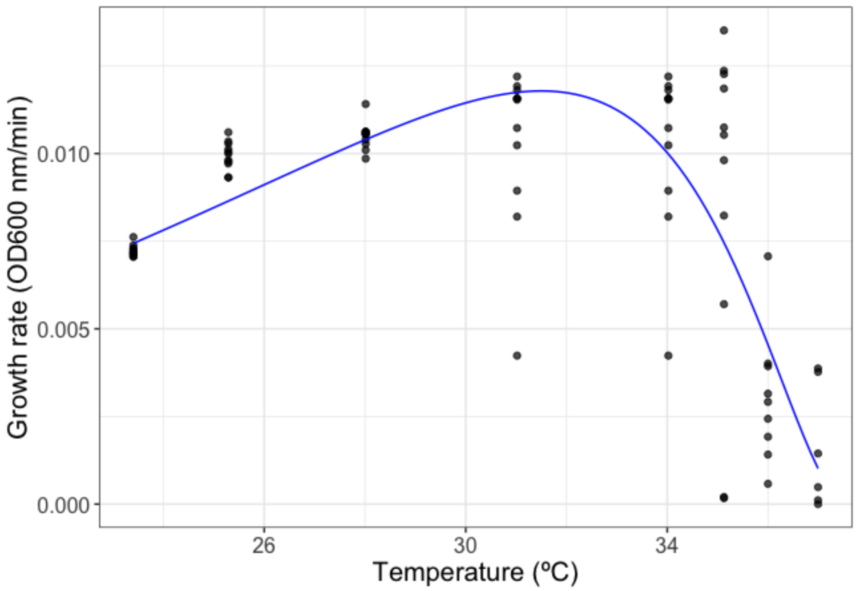
Growth rate (OD600 nm/min)
0.010
0.005
0.000
26
30
34
Temperature (ºC)

AN88

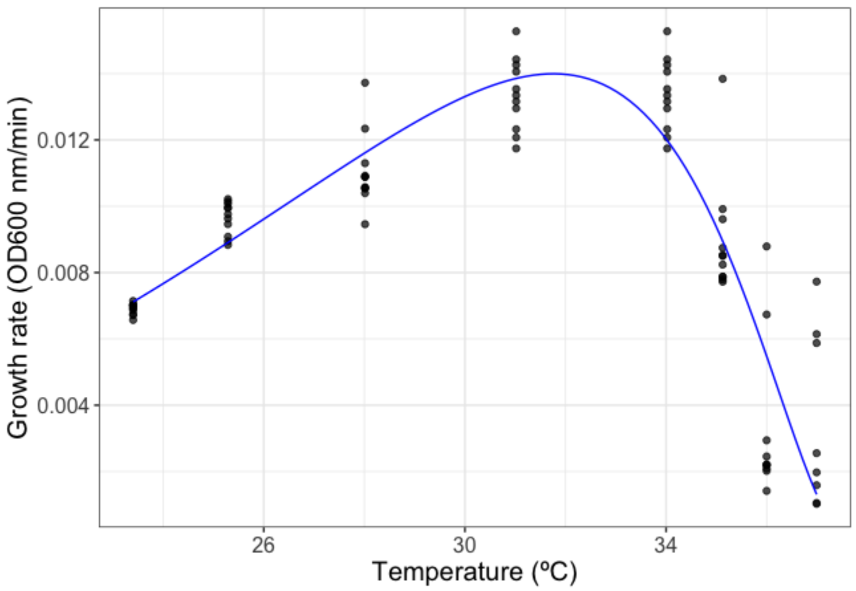
Growth rate (OD600 nm/min)
0.012
0.008
0.004
26
30
34
Temperature (ºC)

AN95

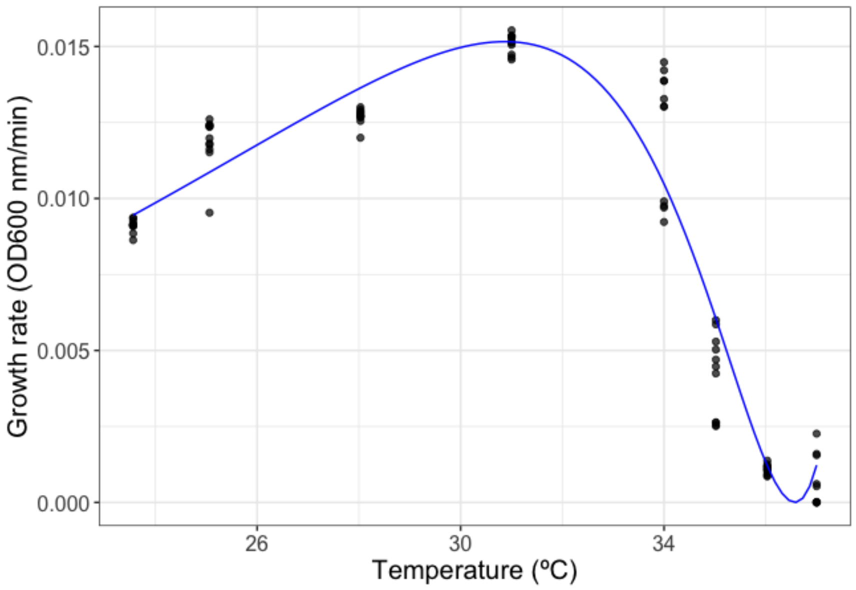
Growth rate (OD600 nm/min)
0.015
0.010
0.005
0.000
26
30
34
Temperature (ºC)

GAS191

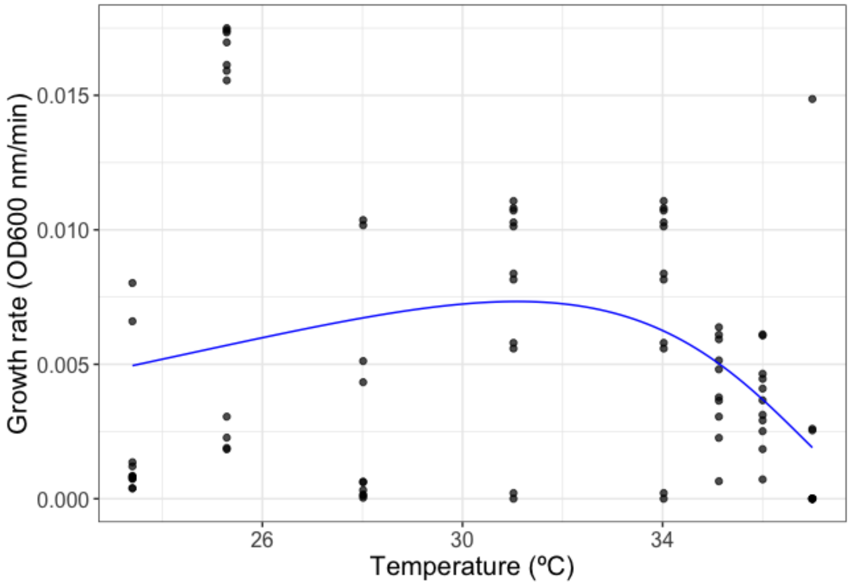

GAS231

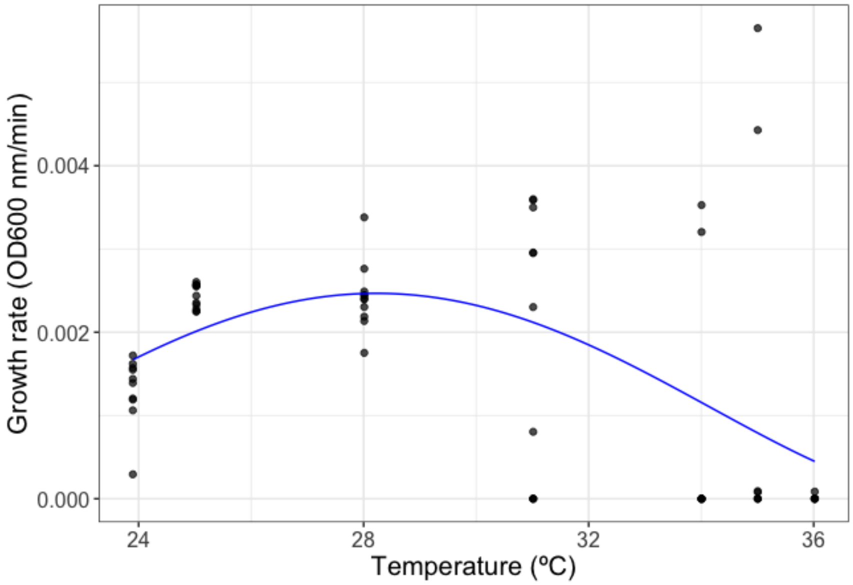

GAS462

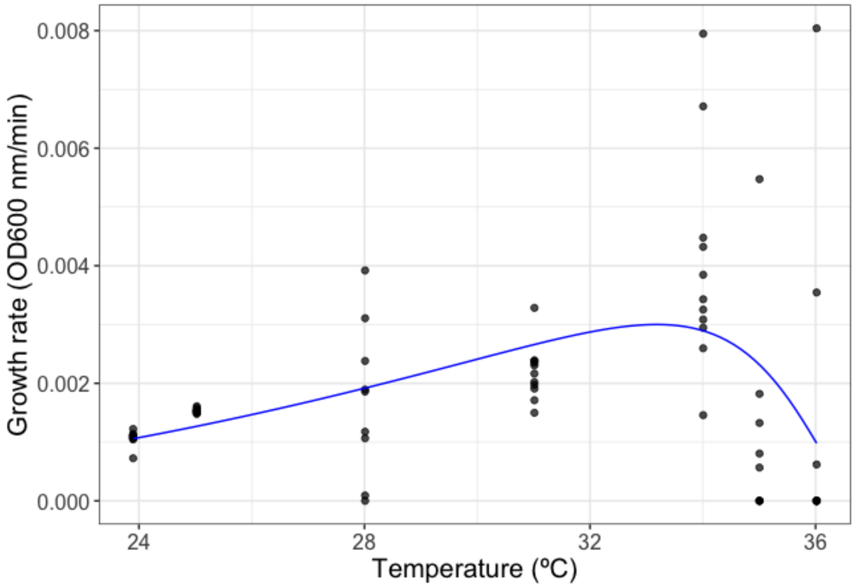

GAS524

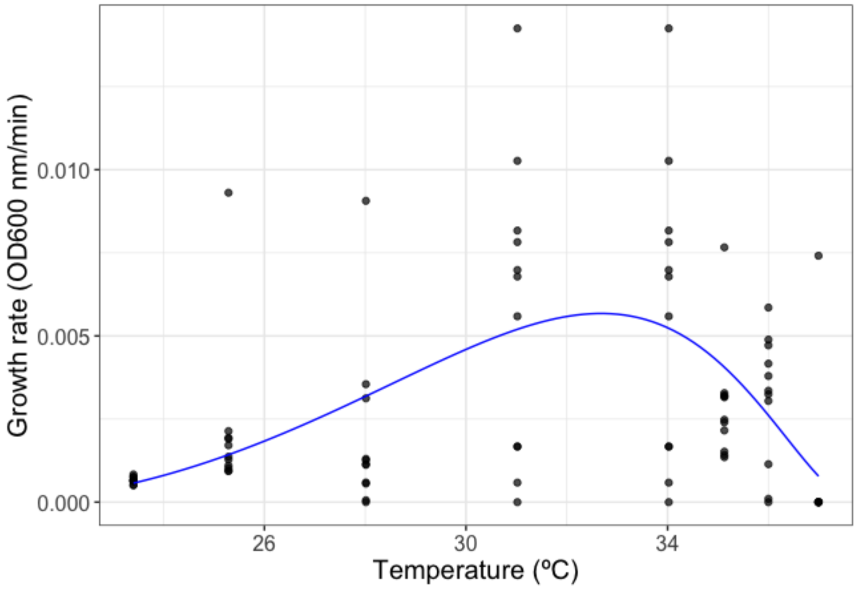

MT12

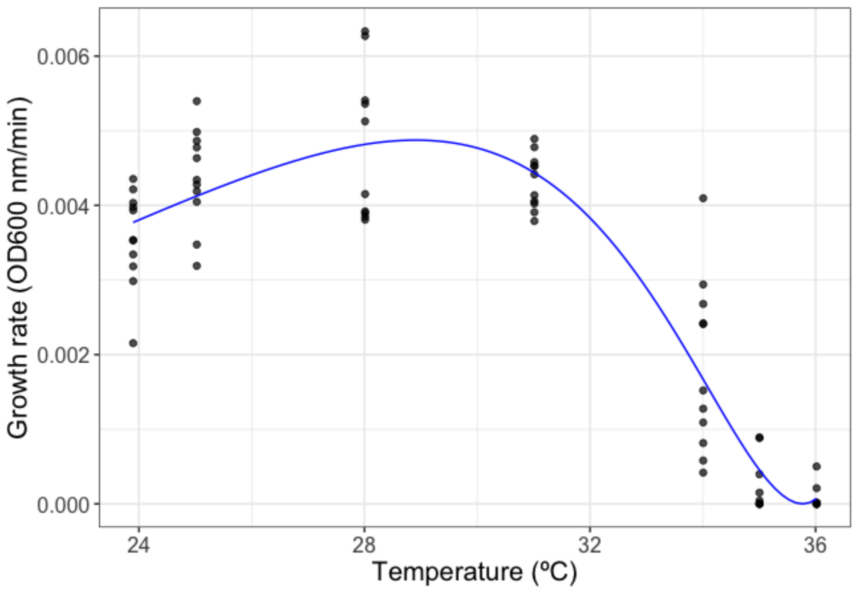
0.006
0.004
0.002
0.000
Growth rate (OD600 nm/min)
24
28
32
36
Temperature (ºC)
