# Supplementary material for: Thermal adaptation of soil microbial growth traits in response to chronic warming
Source: Appl Environ Microbiol. 2023 Oct 25;89(11):e00825-23. doi: 10.1128/aem.00825-23 (PMC10686086; doi:10.1128/aem.00825-23)

Fig S2. Modified Macromolecular Rate Theory (MMRT) model was fitted on data of log transformed growth rate over temperature for each isolate. Temperature sensitivity of growth and optimum growth temperature were estimated from model fits and used for phylogenetic group comparisons.

19YEA23

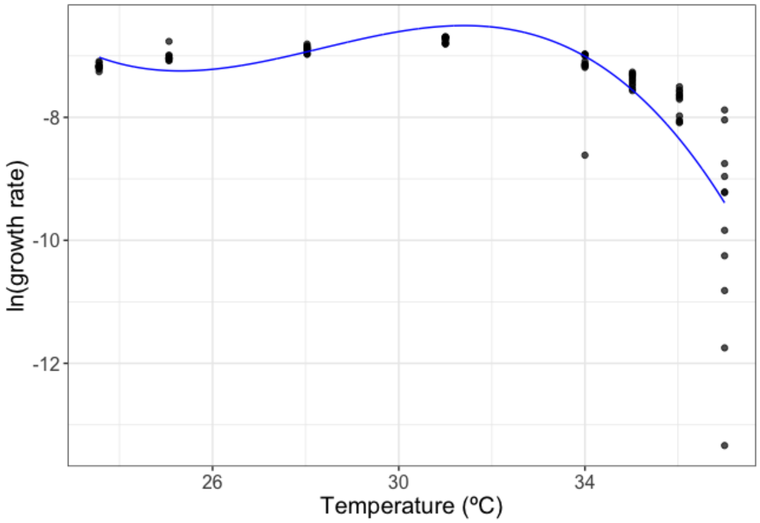

24YEA27

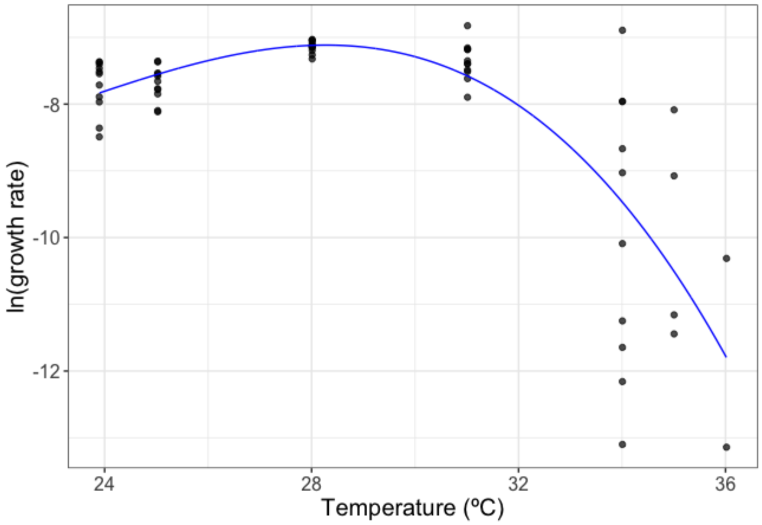

28DA2

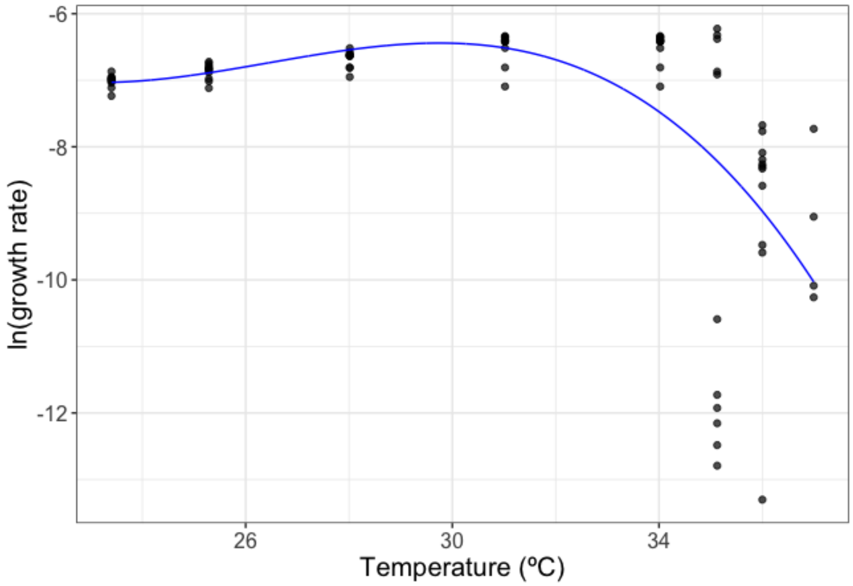

28YEA48

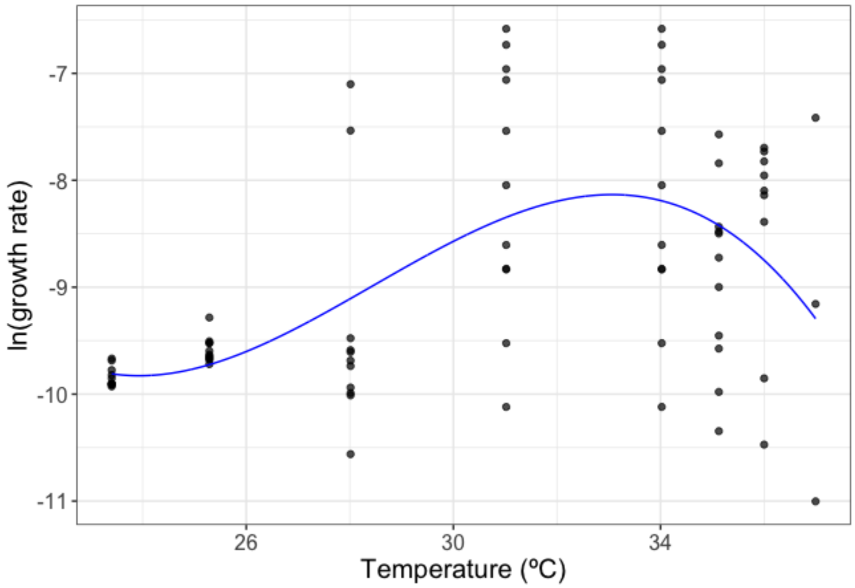

AN5

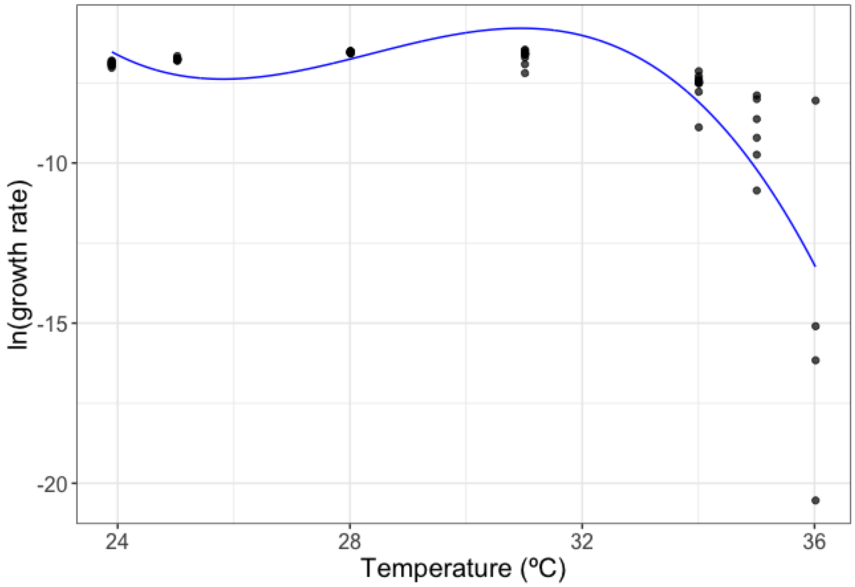

-10
-15
-20
ln(growth rate)
24
28
32
36
Temperature (ºC)

AN6A

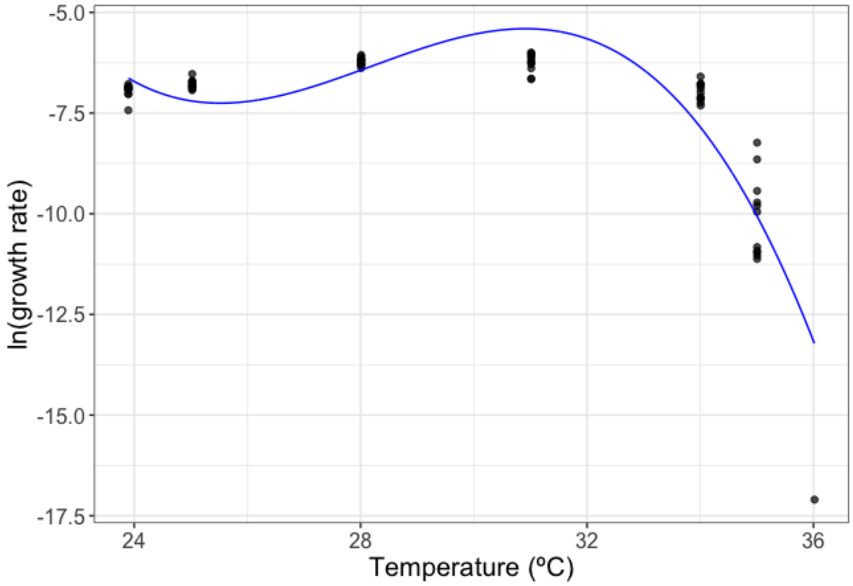

-5.0
-7.5
-10.0
-12.5
-15.0
-17.5
ln(growth rate)
24
28
32
36
Temperature (ºC)

AN63

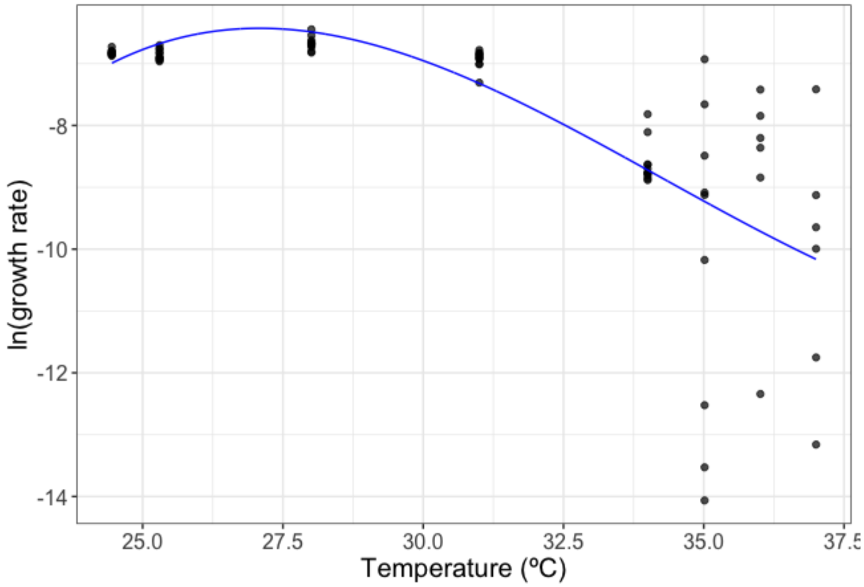

-8
-10
-12
-14
ln(growth rate)
25.0
27.5
30.0
32.5
35.0
Temperature (ºC)

AN64

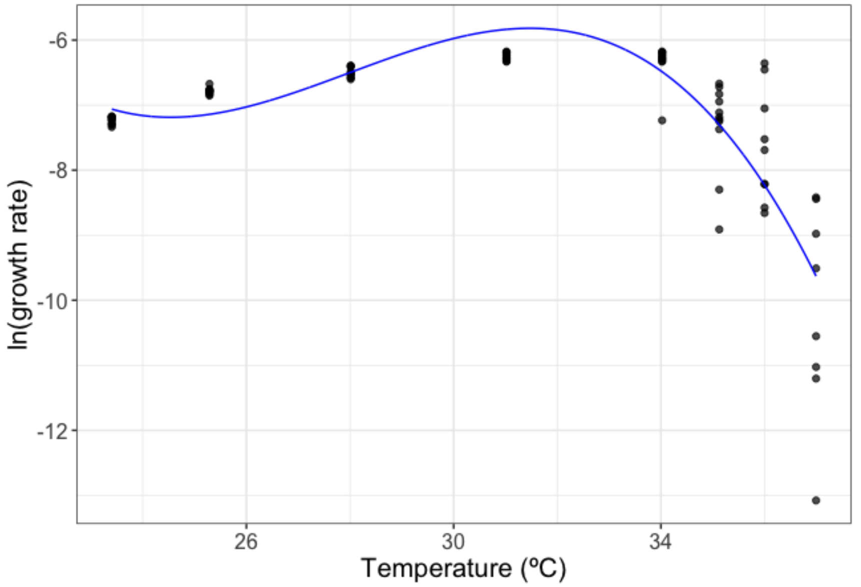

-6
-8
-10
-12
ln(growth rate)
26
30
34
Temperature (ºC)

AN67

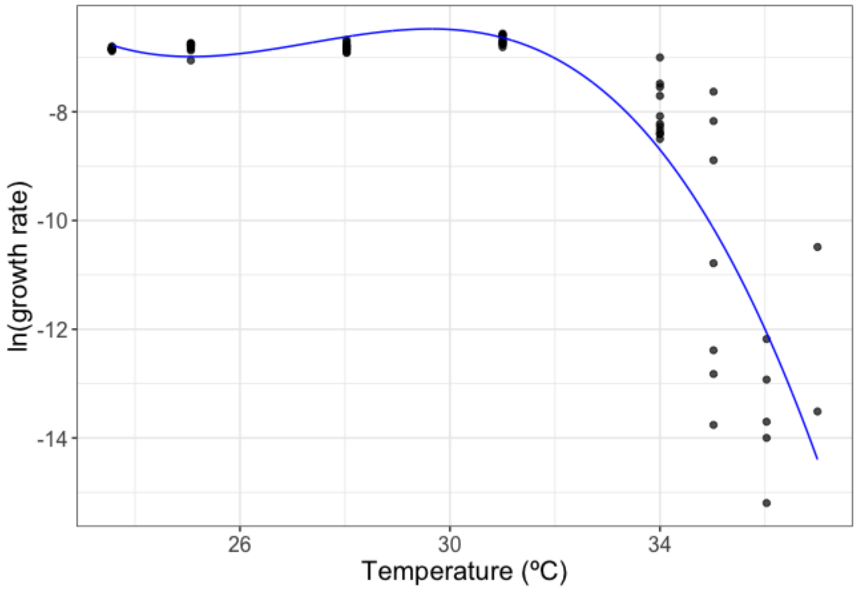

-8
-10
-12
-14
ln(growth rate)
26
30
34
Temperature (ºC)

AN68

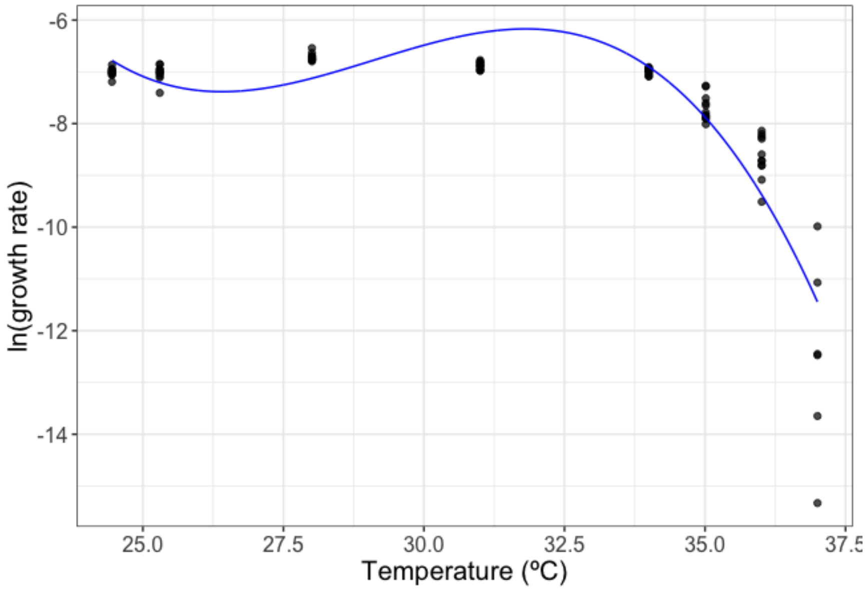

-6
-8
-10
-12
-14
ln(growth rate)
25.0
27.5
30.0
32.5
35.0
37.5
Temperature (ºC)

AN69

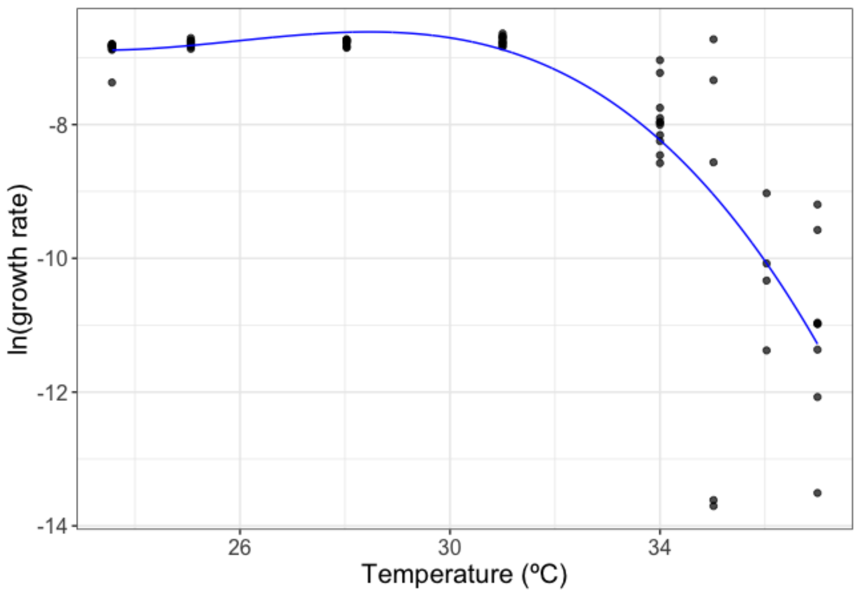

-8
-10
-12
-14
ln(growth rate)
26
30
34
Temperature (ºC)

AN70

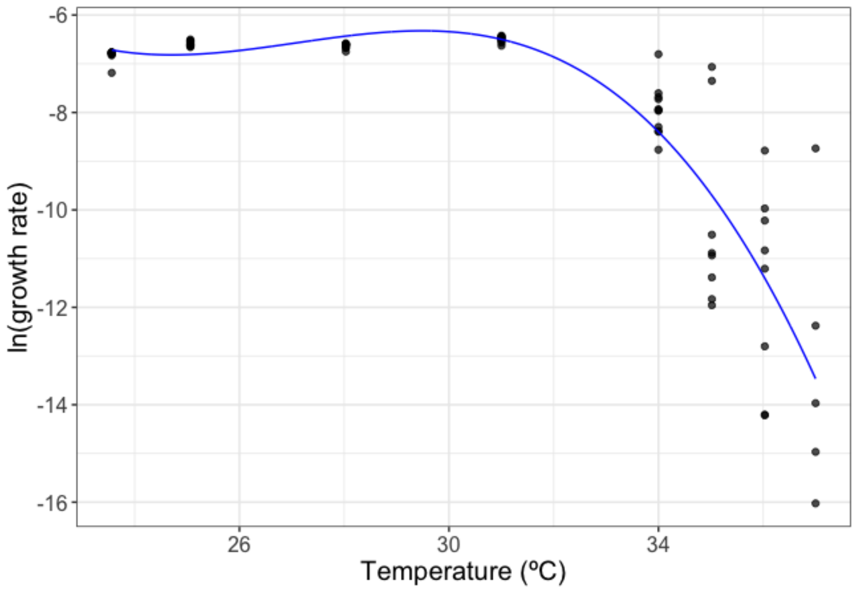

-6
-8
-10
-12
-14
-16
ln(growth rate)
26
30
34
Temperature (ºC)

AN73

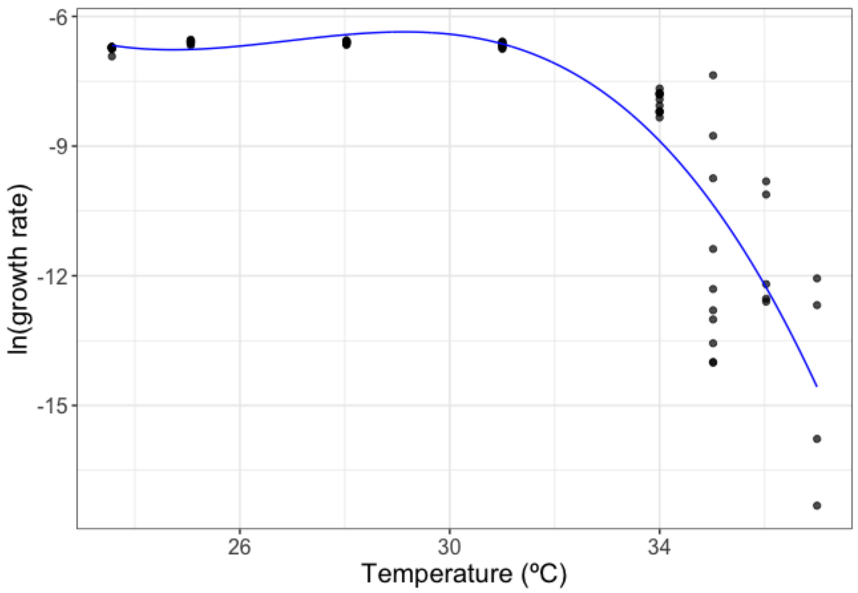

-6
-9
-12
-15
ln(growth rate)
26
30
34
Temperature (ºC)

AN78

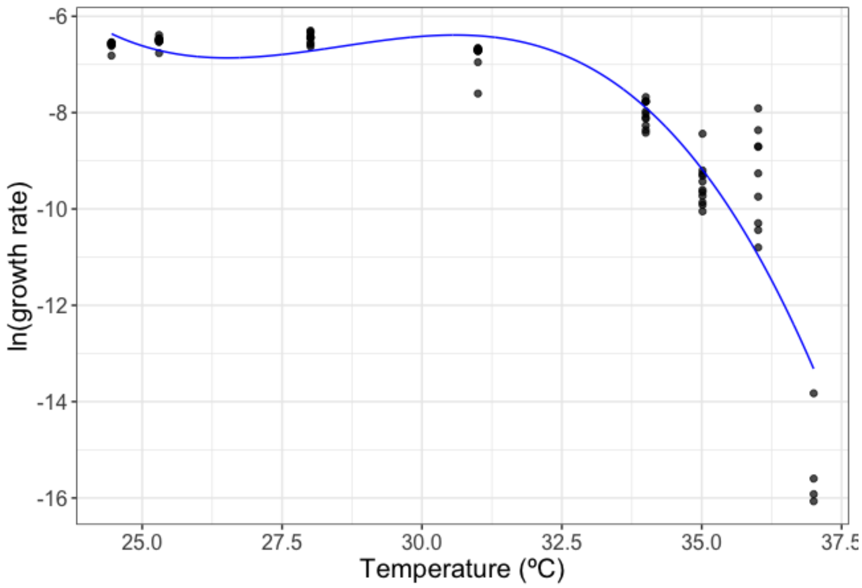

-6
-8
-10
-12
-14
-16
ln(growth rate)
25.0
27.5
30.0
32.5
35.0
37.5
Temperature (ºC)

AN80A

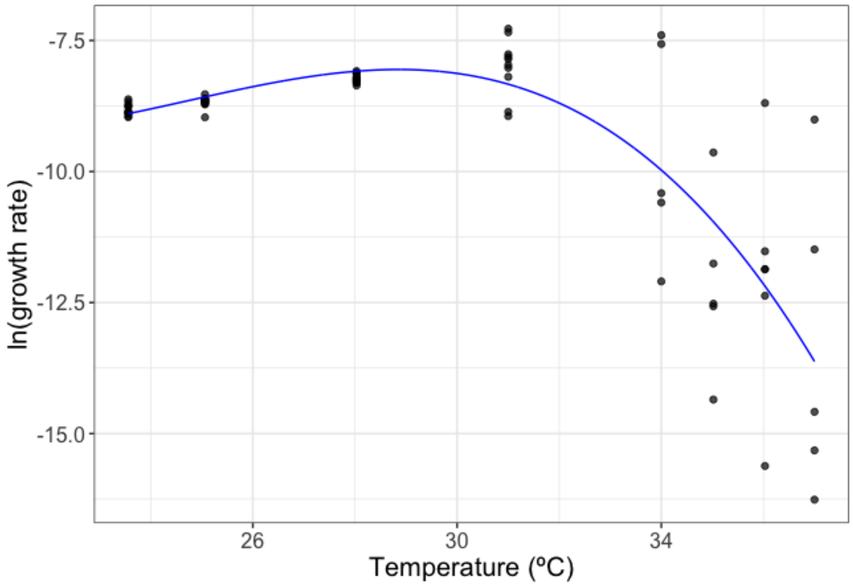
-7.5
-10.0
-12.5
-15.0
ln(growth rate)
26
30
34
Temperature (ºC)

AN83

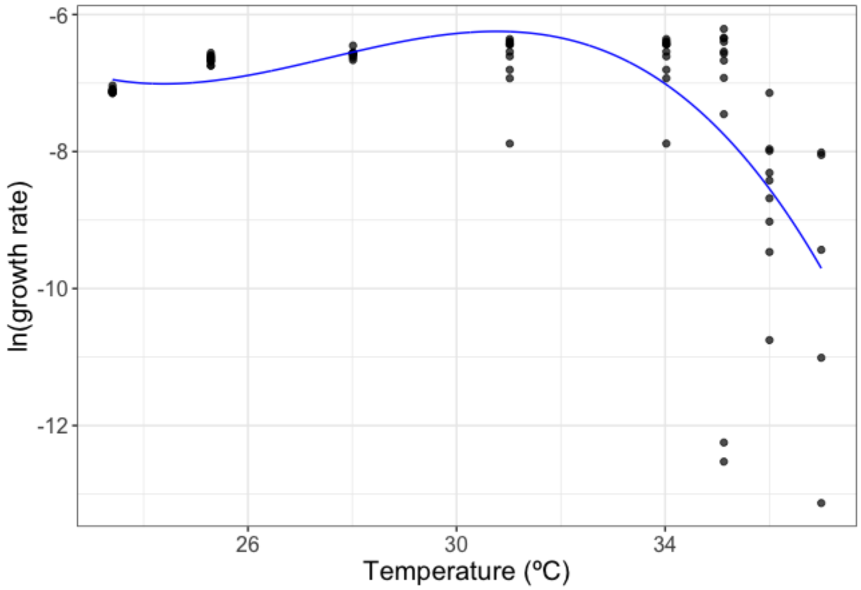
-6
-8
-10
-12
ln(growth rate)
26
30
34
Temperature (ºC)

AN88

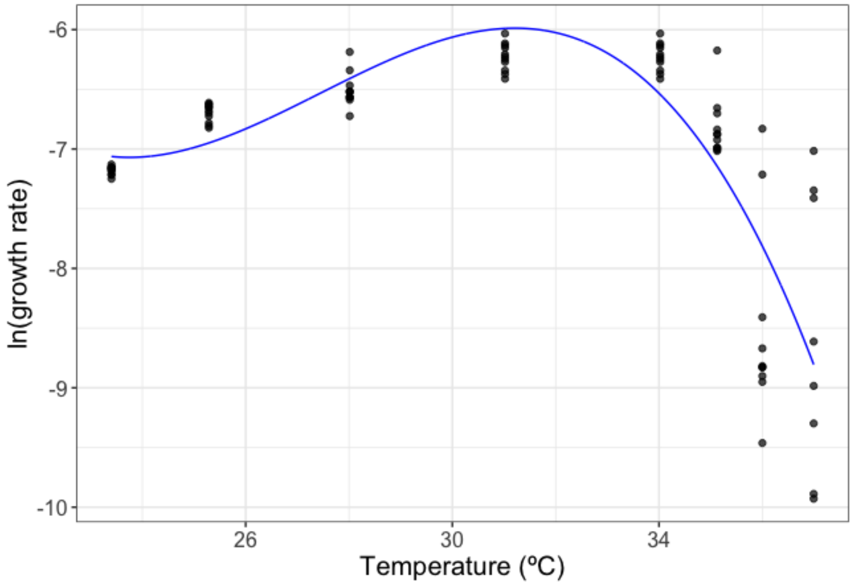
-6
-7
-8
-9
-10
ln(growth rate)
26
30
34
Temperature (ºC)

AN95

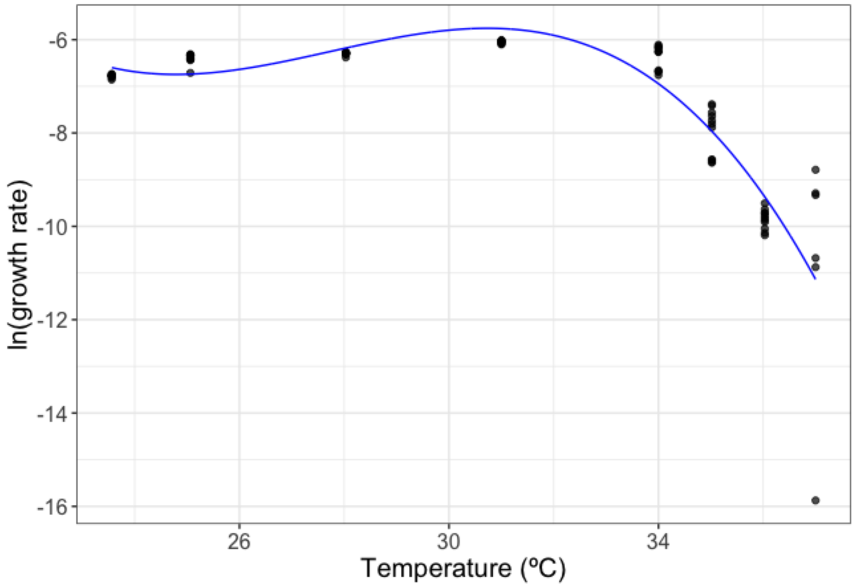
-6
-8
-10
-12
-14
-16
ln(growth rate)
26
30
34
Temperature (ºC)

GAS191

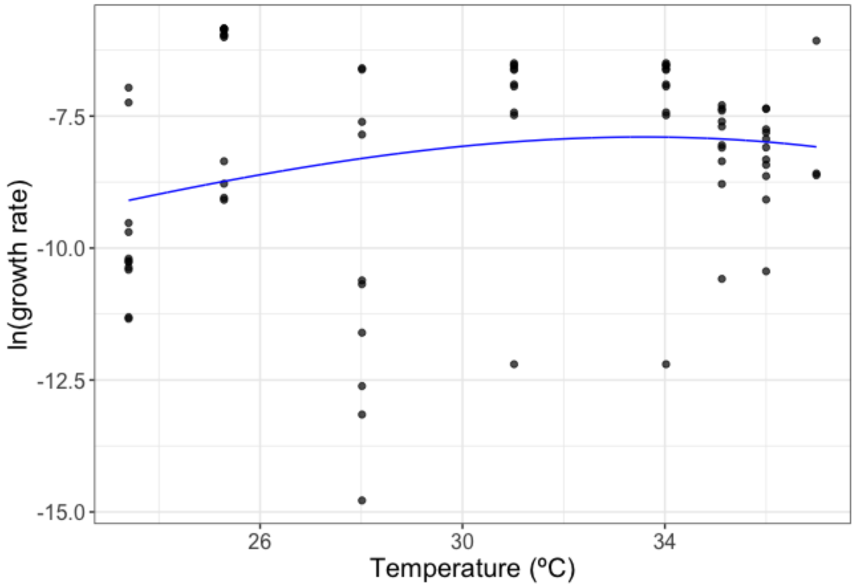

GAS231

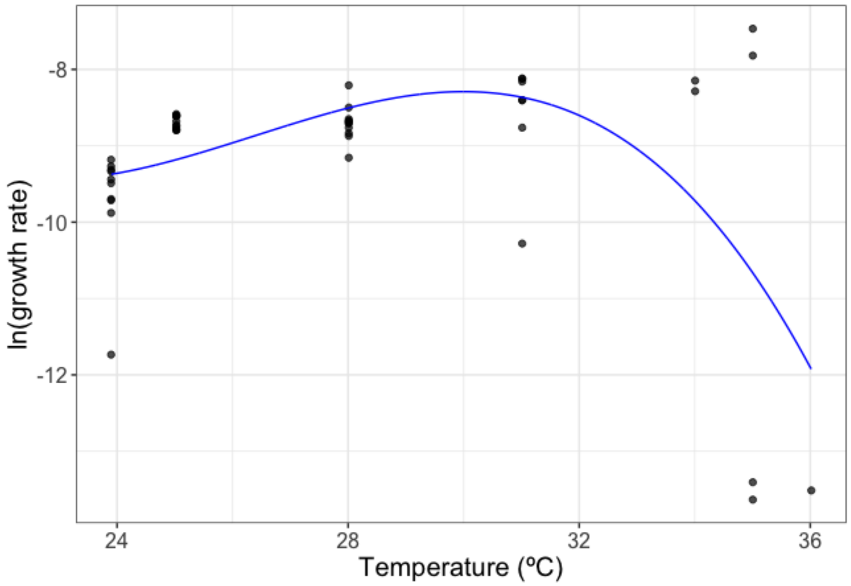

GAS462

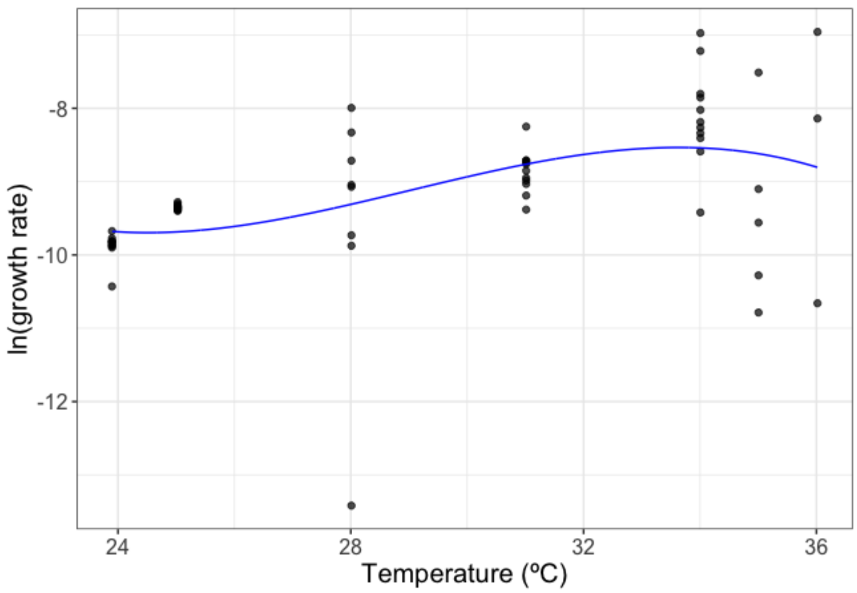

GAS524

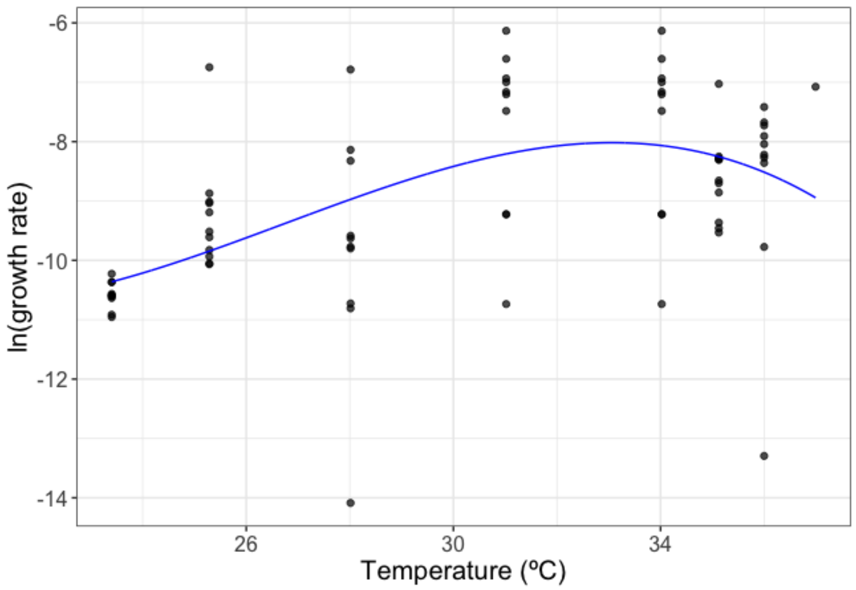

MT12

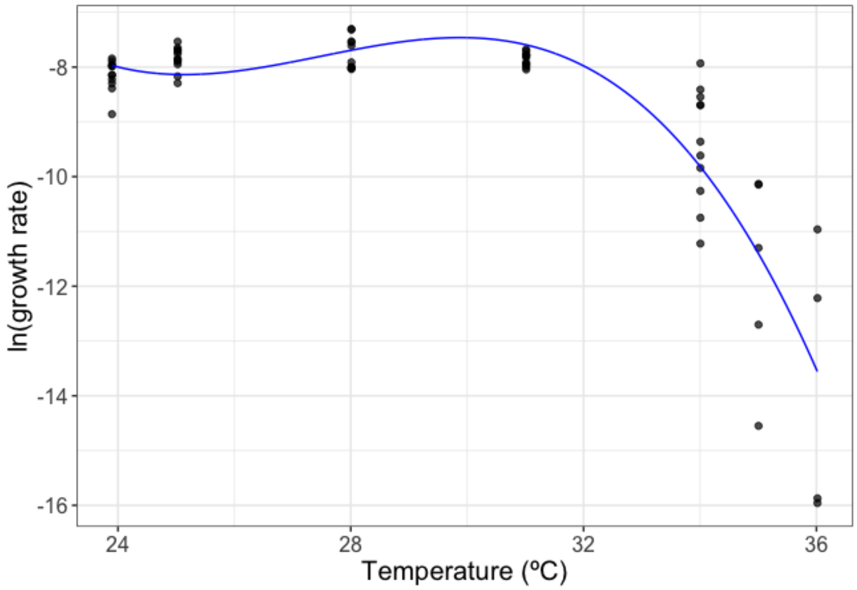

Supplement: Fig. S2 — Macromolecular Rate Theory fits for all isolates. [file aem.00825-23-s0002.pdf]
